# Pharmacokinetic-guided Management of Foslevodopa/Foscarbidopa infusion in *PINK1*-related Parkinson's disease with neuropsychiatric vulnerability

**DOI:** 10.1016/j.prdoa.2026.100487

**Published:** 2026-07-25

**Authors:** Shohei Okusa, Yuki Shimazu, Risano Sakai, Takeshi Akiyoshi, Tomonori Nukariya, Yoshihiro Nihei, Jin Nakahara, Hisakazu Ohtani, Morinobu Seki

**Affiliations:** a Department of Neurology, Keio University School of Medicine, Tokyo, Japan; b Parkinson's Disease Center, Keio University Hospital, Tokyo, Japan; c Center for Parkinson's disease Research, Keio University, Tokyo, Japan; d Division of Clinical Pharmacy, Faculty of Pharmaceutical Sciences, Keio University, Tokyo, Japan; e Department of Clinical Pharmacy, Keio University School of Medicine, Tokyo, Japan; f Department of Pharmacy, Keio University Hospital, Tokyo, Japan

**Keywords:** Continuous subcutaneous infusion, Foslevodopa/foscarbidopa, 3-*O*-methyldopa, L-type amino acid transporter 1, Parkinson's disease, *PTEN-induced putative kinase 1*

Mutations in *PTEN-induced putative kinase 1* (*PINK1*) cause autosomal recessive Parkinson's disease (PD). Although *PINK1*-related PD is generally characterized by a good response to levodopa, patients often develop early wearing-off and levodopa-induced dyskinesia. [1] In addition, higher frequencies of psychotic symptoms have been reported. [2] Here, we report a case of *PINK1*-related PD with neuropsychiatric vulnerability in whom serial pharmacokinetic monitoring of plasma levodopa and 3-*O*-methyldopa (3-OMD) provided clinically useful information for the management of continuous subcutaneous foslevodopa/foscarbidopa (LDp/CDp) infusion.

A Japanese woman developed bradykinesia in her right lower limb at 40 years of age and was diagnosed with clinically established Parkinson's disease (PD) at 42 years, when levodopa therapy was initiated. Wearing-off appeared at 46 years of age, and levodopa-induced dyskinesia emerged at 49 years. Subsequent genetic testing identified a heterozygous *PINK1* variant (c.1023G > A), and the patient was diagnosed with *PINK1*-related PD. Device-aided therapy was considered; however, the patient declined surgical treatment and was therefore followed conservatively.

At 58 years of age, motor complications became difficult to control with oral medication, and device-aided therapy was reconsidered. Levodopa responsiveness remained good (Movement Disorders Society (MDS) -Unified Parkinson's disease Rating Scale (UPDRS) Part III off/on: 65/22 points), and no apparent cognitive impairment was observed. The noise pareidolia test was negative. Based on these findings, continuous subcutaneous LDp/CDp was initiated.

Ten months after initiation of LDp/CDp, the patient was admitted for medication adjustment because of severe anxiety and depressive symptoms during early-morning OFF periods, accompanied by evening-to-nighttime visual hallucinations involving aggressive content and persecutory delusions. On admission, LDp/CDp was administered at 0.25 mL/h during the daytime and 0.17 mL/h at nighttime, and levodopa/carbidopa/entacapone combination tablets were administered every 3 h, six times per day. Under these conditions (total levodopa equivalent dose, 1451 mg/day), plasma levodopa and 3-OMD concentrations and MDS-UPDRS Part III scores were serially assessed on hospital day 2. To estimate the effective central levodopa availability, we calculated a corrected levodopa concentration accounting for competitive L-type amino acid transporter 1 (LAT1) inhibition and by 3-OMD, using receptor-binding theory and the inhibitory constant of 3-OMD (Ki = 56.4 μM). [3] The measurements showed low morning plasma levodopa concentrations corresponding to early-morning OFF symptoms, increased evening-to-nighttime concentrations corresponding to psychiatric symptoms, and a pronounced dissociation between observed and corrected plasma levodopa concentrations associated with markedly elevated 3-OMD levels (Fig. 1).

**Fig. 1:**
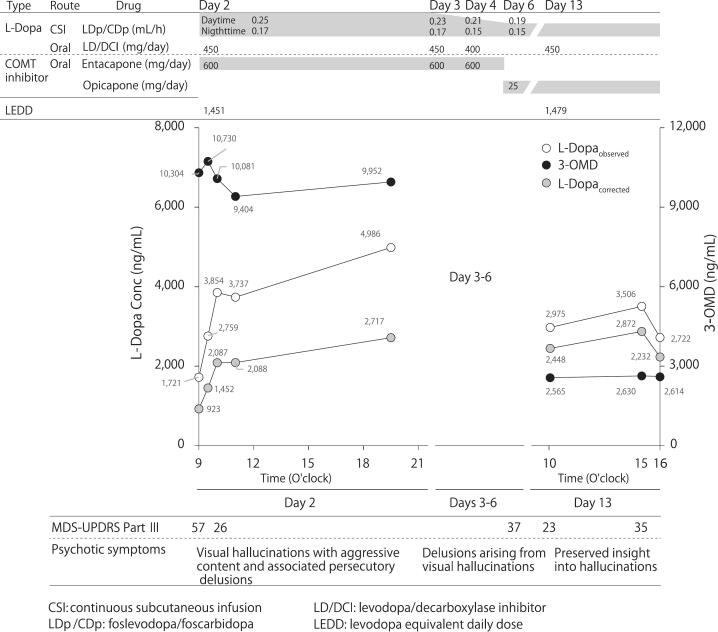
Time-concentration profiles of plasma levodopa and 3-OMD, estimated LAT1 inhibition, and longitudinal changes in motor and psychiatric symptoms.

Based on these findings, oral levodopa/carbidopa was changed from alternating 100- and 50-mg doses to a morning-weighted 100–100–100–50–50–50 mg schedule to improve early-morning OFF symptoms while reducing afternoon-to-nighttime dopaminergic exposure. The catechol-*O*-methyltransferase (COMT) inhibitor was switched from entacapone to opicapone, and the LDp/CDp was reduced to 0.19 mL/h during the daytime and 0.15 mL/h at nighttime. Follow-up plasma measurements on hospital day 13, under comparable conditions (total levodopa equivalent dose, 1479 mg/day), showed a marked reduction in 3-OMD, with stabilization of corrected levodopa concentrations and on state. Psychiatric symptoms also improved: persecutory delusions resolved, and although visual hallucinations persisted, insight was preserved.

To our knowledge, this is the first report of LDp/CDp treatment in a patient with PD carrying a *PINK1* variant. In the present case, visual hallucinations were not present before LDp/CDp initiation; however, the patient's genetic background may have conferred underlying vulnerability to neuropsychiatric symptoms.

These pharmacokinetic measurements provided clinically relevant information in several respects. These pharmacokinetic measurements provided clinically relevant information in several respects. First, although the observed and corrected levodopa concentrations showed similar temporal profiles, correction for estimated LAT1 inhibition by 3-OMD provided additional information regarding effective central levodopa availability. This was particularly relevant when persistent OFF symptoms were observed despite apparently adequate plasma levodopa concentrations, suggesting that high circulating 3-OMD may have limited levodopa transport across the blood-brain barrier. Second, the marked reduction in 3-OMD after switching from entacapone to opicapone supported the pharmacological rationale for sustained COMT inhibition during continuous LDp/CDp infusion. [4] Stabilization of the corrected levodopa concentration paralleled improvements in both motor fluctuations and psychiatric symptoms, suggesting that serial pharmacokinetic monitoring may provide objective support for clinical decision-making. Although corrected levodopa concentrations remained relatively high on Day 13, both motor and psychiatric symptoms improved. This may reflect the combined effects of treatment adjustments, including redistribution of oral levodopa dosing, reduction of LDp/CDp infusion across the day and night, and optimization of COMT inhibition, which together altered the temporal pattern of dopaminergic exposure. These findings suggest that clinical tolerability may depend not only on estimated effective levodopa availability, but also on the timing and fluctuation of dopaminergic stimulation. Because both treatment modifications were implemented simultaneously to optimize therapy while maintaining an appropriate levodopa equivalent daily dose, the individual contribution of each intervention to the reduction in 3-OMD levels and clinical improvement cannot be determined.

This case has practical implications for managing LDp/CDp therapy in patients with neuropsychiatric vulnerability. Because the patient declined surgical device-aided therapies, LDp/CDp represented a clinically relevant alternative. Although conversion back to oral levodopa therapy should be considered in patients who develop psychosis during LDp/CDp treatment, our patient experienced substantial improvement in disabling morning OFF symptoms and preferred to continue infusion therapy. Extended-release oral levodopa formulations (e.g., Crexont and Rytary) may represent alternative therapeutic options; however, these formulations are not currently available in Japan and therefore were not therapeutic options for our patient. Although continuous levodopa delivery may stabilize plasma levodopa exposure and improve motor fluctuations, it may also unmask or worsen psychiatric symptoms in vulnerable individuals. [5] Thus, careful patient selection, pre-treatment psychiatric assessment, gradual titration, and prompt dose adjustment are essential. A key implication is the potential utility of serial pharmacokinetic monitoring for individualized LDp/CDp management. Whereas hallucinations or delusions are often managed empirically by reducing dopaminergic medications or adding antipsychotic agents, serial levodopa and 3-OMD measurements provided objective information on the balance between motor benefit and psychiatric tolerability. The corrected levodopa concentration, adjusted for estimated LAT1 inhibition by 3-OMD, served as an exploratory index of this balance and supported treatment adjustments, including LDp/CDp dose reduction and replacement of entacapone with opicapone. After these changes, 3-OMD levels decreased, corrected levodopa availability stabilized, and persecutory delusions resolved. The remaining visual hallucinations were mild, accompanied by preserved insight, and considered tolerable by the patient; therefore, antipsychotic therapy was not initiated. These findings suggest that pharmacokinetic-guided management may help optimize LDp/CDp therapy beyond initiation, particularly when motor and psychiatric symptoms require careful balancing.

Importantly, this case does not suggest that LDp/CDp is superior to oral levodopa therapy or other treatment strategies. Rather, it suggests that, when LDp/CDp is selected for patients with neuropsychiatric vulnerability, serial pharmacokinetic monitoring may provide a useful framework for individualized long-term management. Such monitoring may be particularly relevant during continuous 24-h LDp/CDp infusion, in which sustained levodopa exposure and 3-OMD accumulation may influence both motor response and psychiatric tolerability.

In summary, this case represents the first report of LDp/CDp use in a patient with PD harboring a *PINK1* variant and suggests that pharmacokinetic-guided adjustment of LDp/CDp may help improve motor symptoms while reducing neuropsychiatric adverse effects to a clinically tolerable level in selected patients. Future studies comparing this approach with alternative treatment strategies, including extended-release oral levodopa formulations where available, are warranted.

## Ethical compliance statement

Ethical approval for this study was obtained from the institutional ethics committee of Keio University School of Medicine (approval number #20251194), and informed consent was obtained from this participant. Verbal informed consent for publication was obtained from the patient and formally documented in the medical record. We confirm that we have read the Journal's position on issues involved in ethical publication and affirm that this work is consistent with those guidelines.

## Author Declaration

Clinical Parkinsonism & Related Disorders is committed to proper scientific conduct and the protection of animal and human research subjects. Submission of this manuscript implies compliance with the following ethical requirements. Please affirm that you are representing all of the authors in stating compliance with these policies by checking the box at the end of this section.

1. Studies with human subjects must have been conducted in accordance with the Declaration of Helsinki. All persons must have provided informed consent prior to being included in the study.
2. Studies with animal subjects must have been conducted in accordance with the Guide for the Care and Use of Laboratory Subjects as adopted by the US National Institutes of Health and/or according to the requirements of all applicable local, national and international standards.
3. Protocols with animal or human subjects must have been approved by the relevant local committee(s) charged with ensuring subject protection. Studies that entail pain or distress will be assessed in terms of the balance between the distress inflicted and the likelihood of benefit.
4. The authors declare that the manuscript is original, that it is not being considered for publication elsewhere, and that it will not be submitted elsewhere while still under consideration for Clinical Parkinsonism & Related Disorders or after it has been accepted by Clinical Parkinsonism & Related Disorders.
5. All authors have seen and approved the manuscript in the form submitted to the journal. The authors declare that they have conformed to the highest standards of ethical conduct in the submission of accurate data and that they acknowledge the work of others when applicable.
6. All sources of financial support for the work have been declared in the Acknowledgements section of the manuscript. Any additional conflicts of interest must also be declared. Please include declarations of any consultancy or research funding received from relevant companies from three years prior to performance of the research until the time of manuscript submission. If the research is supported by internal funds, that should be stated as well.

To indicate compliance with the preceding declaration and that you have obtained agreement from all of the authors of this paper to declare their compliance as well, please place an x here: **X.**

In cases of uncertainty please contact an editor for advice.

## CRediT authorship contribution statement

**Shohei Okusa:** Writing – original draft, Resources, Project administration, Methodology, Investigation, Formal analysis, Data curation, Conceptualization. **Yuki Shimazu:** Resources, Investigation. **Risano Sakai:** Investigation, Formal analysis, Data curation. **Takeshi Akiyoshi:** Writing – original draft, Software, Resources, Methodology, Formal analysis, Data curation. **Tomonori Nukariya:** Resources. **Yoshihiro Nihei:** Supervision. **Jin Nakahara:** Supervision. **Hisakazu Ohtani:** Writing – review & editing, Supervision. **Morinobu Seki:** Writing – review & editing, Supervision, Resources, Methodology, Investigation, Data curation, Conceptualization.

## Funding

All authors report no financial relationships and no conflicts of interest with commercial interests.

## Declaration of competing interest

The authors declare that they have no known competing financial interests or personal relationships that could have appeared to influence the work reported in this paper.
